# Surface structuring of glass with submicrometer features using selective laser etching

**DOI:** 10.1038/s41598-025-27241-0

**Published:** 2025-11-10

**Authors:** Axel Günther, Xiaowei Wang, Wolfgang Kowalsky, Bernhard Roth

**Affiliations:** 1 Institute of High Frequency Technology, Technical University Braunschweig, 38106 Braunschweig, Germany; 2 Hannover Centre for Optical Technologies, Leibniz University of Hannover, 30167 Hannover, Germany; 3 Photonics, Optics and Engineering – Innovation Across Disciplines, Cluster of Excellence PhoenixD, 30167 Hannover, Germany

**Keywords:** Selective laser etching, Optical waveguides, Integrated optics, Optical physics, Surface patterning

## Abstract

Selective laser etching (SLE) is a procedure to create customized micro-fluidic or micro-mechanical structures in glass. We aim to utilize SLE for optics production as well and report on detailed investigations of the glass structuring process by variation of stage speed, laser power, polarization and repetition rate of the laser to reduce the surface roughness to optical quality without any additional process steps. We show that a surface roughness of $\approx$30 nm can be achieved which is sufficient to fabricate optical components or integrated optical structures even for the visible wavelength range. Our results enable the fabrication of free standing micro-structures with a feature size of less than 1 µm in fused silica using SLE and KOH as etchant which enables the fabrication of optical gratings.

## Introduction

Laser assisted material processing was well investigated during the last decades and is established in a broad field of applications nowadays. Depending on the used systems and materials it can be used for ablation, micro-structuring, welding, soldering or for additive manufacturing, among others^1–11^. Generally, a broad field of materials can be modified and structured by using laser pulses^7,8,10^. In this work, we focus on selective laser etching (SLE) which enables the fabrication of 2- or 3-dimensional optical structures on the surface or within glass and sapphire substrates, among others. Here, an ultrafast laser pulse is absorbed in the transparent media at the focal point due to nonlinear effects. This generates a free charge carrier plasma inside the material which leads to the ionization of the silica. The thereby induced micro-explosions densify the material around the focal spot which leads to decreased bridging angles of $SiO_4$ units. This distortion results in an increased reactivity of the oxygen atoms due to the distorted configuration of the oxygen’s valence electrons. That, in turn, implies that the irradiated areas show a significant higher etch-rate than the unexposed ones^12^. SLE is under investigation since nearly two decades and is actually used for micro-mechanics, micro-fluidics and customized micro-optics, respectively^13–20^. Due to the availability of materials with high optical transparency, chemical resilience against most organic solvents or distinct bio-compatibility the technique is applicable to realize optical sensors for bio detection or gas and mechanical sensing, for example^21–26^ which were realized with other techniques than SLE. The influence of different etchants such as pottasium hydroxide (KOH), hydrofluoric acid (HF), sodium hydroxide (NaOH) or phosphoric acid mixture ($H_2SO_4$ and $H_3PO_4$) has been investigated as well which is necessary to process different types of glass, i.e. fused silica, borosilicate glass or sapphire.^27,28^. In previous studies the surface roughness was measured and values of $S_a \ge 100\,nm$ were achieved. The surface roughness can be reduced even further with an additional annealing step. Hereby, the roughness can be reduced to $S_a \le 10\,nm$^12,29,30^. Recent works in SLE are focusing on manufacturing of macroscopic micro-optics and micro-mechanical structures with a size of a few hundred µm or a few mm^3,17,19,31,32^. There are further techniques to structure transparent media such as Laser induced backside wet etching (LIBWE) where the laser beam is transmitted through the glass completely and absorbed linearly by a neighboring material. This forms a plasma at the boundary resulting in local ablation or material transfer towards the glass, respectively^33^. Due to the linear absorption-based process heat will be transferred to the material which is avoided during SLE. Otherwise it might lead to additional internal stress resulting in cracks, especially at corners with a sharp angle. On the other hand, LIBWE achieves a better surface roughness due to the smaller etching rate per pulse^33,34^. In this work we focus on the fabrication of integrated optics in glass by SLE with reduced surface roughness displaying optical quality at the level of $S_a < \lambda /10$ without any post processing step which is used as threshold for commercially flat-optics and commonly likewise to allow low-loss waveguiding. Additionally, we investigate the integration of further optical structures as diffractive optical element. Such elements, i.e. gratings, have been fabricated in glass earlier but only by using HF as etchant^35–40^. We were able to fabricate free standing features with a width of less than 1 µm with SLE using KOH.

## Methods

### SLE process

**Fig. 1:**
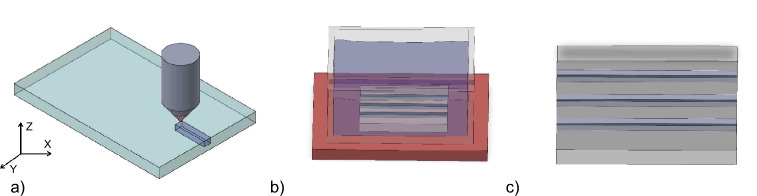
**a** Scheme of required process steps for selective laser etching. At first, **a-1** the fused silica is structured by the fs-laser. Subsequently, **b** the structured substrate is placed into a KOH-bath which is heated to 90 $^{\circ }$ C. Finally, **c** the substrate is finished by flushing with distilled water.

The SLE process relies on two main steps. First the designed structure needs to be transferred to the glass using a fs-laser. In our work, we used fused silica (microscope slides, Micro to Nano BV, Netherlands) as sample material, especially due to its ability to be etched by KOH. The Laser Nanofactory system (Femtika, Lithuania) featuring a 10W Pharos laser (Light Conversion, Lithuania) was used with an 20x objective (NA=0.4, Nikon, Japan) to structure the glass samples. Subsequently, the modified substrate is placed in KOH with a concentration of 10 mol/l which is heated up to $90^{\circ }$ C. The etching duration depends on the depth of the modification and the etching speed which is $\approx 100\,\mu m/h$ for the given combination of fused silica and KOH^15^. An advantage of this material combination is the high selectivity of $\approx 1000$^41^, which defines the ratio of modified and unmodified material etching rates and enables the fabrication of embedded structures. The process used in this work is depicted in Fig. 1.

The SLE process is energy-dependent with a certain ablation or damage threshold of fused silica. For our parameters, we achieve a maximum peak power of $\approx 2\,J/{cm^2}$ at a beam diameter of 5 µm, a pulse energy of $E_{Pulse} = 400$ nJ and a pulse duration of $\tau = 180\,$fs. This indicates that the process proceeds below the threshold.^42^. The beam diameter was estimated by measuring the diameter of single etched spots on the surface, behaving as shown in Fig. 3a. The applied energy leads to a local modification of the fused silica enabling the reactant to etch the modified area. Therefore, each volumetric structure inside the fused silica which is to be etched requires contact to the surface. Hereby, the surface roughness depends mainly on the pulse energy, size and overlap of the focus spots set next to each other, respectively, the latter being controlled by the writing speed. The relevant parameters are thus pulse energy, stage speed, repetition rate and hatching and slicing which defines the distance between each written line in the xy-plane and the z-direction, respectively. Hereby, the z-axis correlates with the optical axis. The investigation of the surface roughness was done using an optical profilometer (S neox, Sensofar metrology, Barcelona) featuring confocal microscopy and white light interferometry, among others. For the measurement, a magnifying objective (150x, Nikon) was used providing a field of view of $\approx$ 150 µm x 150 µm.

### Characterization setup

**Fig. 2:**
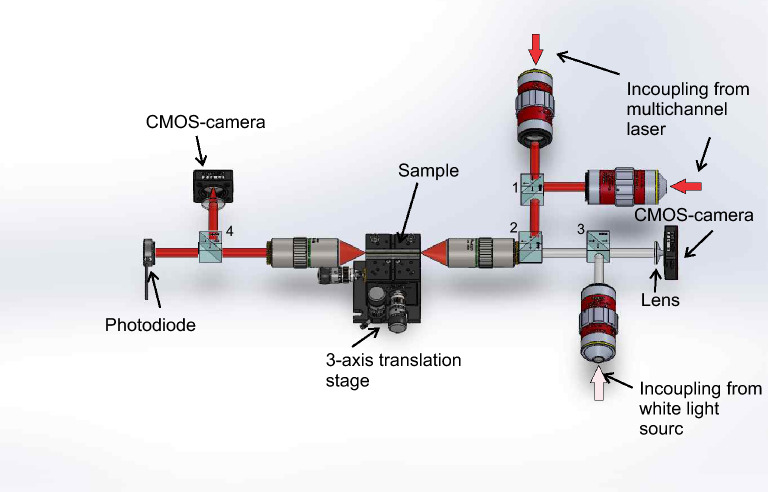
Setup used to couple the light into the waveguide and perform first attenuation measurements. The system consists of a multichannel fiber coupled laser source (L6Cc, Oxxius, France) offering 405 nm, 450 nm, 520 nm, 561 nm, 633 nm and 785 nm which is connected to two objectives collimating the beams. Subsequently, the beams are combined by a dichroitic beam splitter (1). The further beam splitters (2–4) have a splitting ratio of 50:50. An additional white light source is attached enabling spectroscopic measurements and illuminating the sample. The light is focused onto the sample using an objective with a working distance of 20 mm (20X Mitutoyo plan apochromat objective). The same objective is used to collect the transmitted light on the opposite side of the sample. Here, the light is splitted and directed to a camera observing the end facet of the waveguide and a photo diode (S120C, Thorlabs, Germany).

The structured and etched channels were filled with OrmoCore (micro resist technology, Berlin, $n_{\lambda =633}$=1.56) as core material, whereas fused silica has a refractive index of $n_{fused silica}=1.46$ at $\lambda =633\,$nm and serves as cladding. Subsequently, the remaining layer was removed by doctor blading. The waveguide was finalized with a UV-curing step and imaged with the setup depicted in Fig. 2.

## Results and discussion

**Fig. 3:**
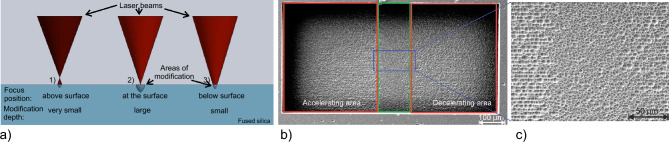
**a** Scheme of the determination of the focal dimension. The three positions depict the z-location of the laser focus in comparison to the glass surface and the corresponding modified area. **a-1** The modification of the material starts already when the position of the beam focus is located above the surface level. **a-2** If the beam focus is on the surface level the applied energy leads to the largest modification width and decreases again if the focus is located below the surface (**a-3**). **b** SEM image of a single etched box with a total length of 1 mm depicting the effects of acceleration and deceleration being visible through the different patterning at the left and right end (in the red boxes) of the structure compared to the center (in the green box), where the stage speed is at a sufficient level to achieve a good surface roughness. Outside of this region, the roughness values are increasing to a few hundred nanometers, which will lead to high scattering losses. To avoid these effects and realize more homogeneous surfaces, the box length was increased to 5 mm for further experiments. The writing direction of the laser was along the long axis of the box which results in a smoother surface compared to a structuring along the short axis of the box. **c** Enlarged image of the central area of the box visualizing the influence of the writing speed on the surface roughness.

The same approach is used to create microstructures with feature sizes less then the spot size by increasing the hatching, which enables the fabrication of gratings or walls in between micro-fluidic or optical channels with a thickness of less than 1 µm, respectively. The focus spot itself shows an elliptical shape which was determined by modifying straight lines in fused silica at different depths with step sizes in z-direction of 1 µm, beginning with the first detectable modification of the glass surface. Hereby an effective average size of the focal spot of 13 µm in z-direction and 5 µm diameter in the x-y plane at the position of the maximum width was measured after etching. The extension of the focal spot in z-direction means, that a modification with contact to the surface can be achieved by locating the focal spot up to 13 µm below the surface. The width of the modification can also be increased if more energy is applied at a single location which appears at the edges of the structure where the stages are accelerating and decelerating and thus illuminating the same spot for longer times. Here a spot diameter of 8.75 µm ± 0.3 µm was measured by focusing the laser on the same position for a few seconds. The etching depth is different from the spot size in z-direction because the modification starts on the surface if the threshold light fluence for material modification is reached there. This parameter was investigated by writing single lines along the x-axis while the focal position along the optical axis was changed by 1 µm per line. Hereby, the smallest etching depth of 0.6 µm was observed when the position of the focus spot is slightly above the surface. The deepest single line was measured with 2.8 µm where the focus was located 13 µm below the surface. At this position, the light fluence at the surface was larger than the modification threshold even if the smallest focal width was located inside the glass. The next line which was inscribed 1 µm deeper led to a modification inside the glass without contact to the surface, where the subsequent etching could not take place. It was not possible to observe the real focal position above or beneath the surface, though a correlation between the focal position and the modification depth is depicted in Fig. 3a. The measurements were performed after 2h of etching at $90^{\circ }$ C in KOH which allows a removal of $\approx$ 200 µm of material in fused silica if a larger volume was modified in vertical direction. Due to the high selectivity of $\approx 1000$, by using fused silica and KOH, a negligible layer of unexposed material with a thickness of 0.2 µm is removed^15^. This feature enables the etching of more complex buried structures.

### Investigation of the surface roughness

To investigate the effect of different process parameters on the surface roughness, multiple boxes with dimensions of 5 mm x 0.5 mm x 0.1 mm [length x width x depth] were etched. Hereby, the ground layer and the surrounding and intermediate walls were modified by the laser. The latter structures are necessary to enable an easy removal of the exposed structure from the substrate. The box size was chosen because experiments showed that the acceleration and deceleration regions of the translation stages have a significant effect on the surface quality, as visualized in Fig. 3b and c. These boxes and other structures were fabricated by scanning the sample with the laser beam line by line. The most important parameters here are hatching and slicing describing the distance between each line in the x-y plane and the z-direction, respectively. These parameters have been investigated in detail in the range from 0.1 µm to 10 µm. The best results regarding surface roughness were achieved with a hatching and slicing of 1 µm with an average laser power of $P_{avg}=250\,$mW and a laser repetition rate of 700 kHz. More detailed information regarding the parameter study are presented in the supplementary material (Supplementary Fig. 1). For the optimization, the process parameters were investigated as given in Table 1. Hereby, the pulse energy was increased in steps of $\approx$ 20 nJ, the stage speed by 1 mm/s and the repetition rate by 100 kHz, respectively.

**Table 1:** Investigated process parameter to optimize the surface roughness.

	Min.	Max.	Best
Energy [nJ]	300	450	350
Speed [mm/s]	15	20	15
Pulse distance [nm]	5	40	20
Spacing [µm]	0.1	10	1
Rep. rate [kHz]	600	1000	700

**Fig. 4:**
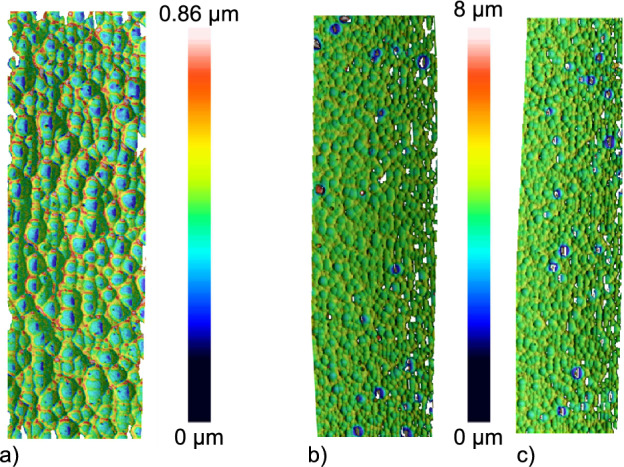
Surface topographies structured with a repetition rate of 700 kHz, pulse energy of 350 nJ and writing speed of 20 mm/s. The measured average surface roughnesses $S_a$ and spatial frequencies $S_{al}$ with the corresponding polarizations are **a** $S_a=30\,nm$ and $S_{al}=640\,nm$ with a parallel polarization, **b** $S_a=65\,nm$ and $S_{al}=700\,nm$ with a perpendicular polarization and **c** $S_a=78\,nm$ and $S_{al}=800\,nm$ with a circular polarization. Hereby, $S_a$ gives the average peak-to-valley value and $S_{al}$ the spatial frequency value averaged over all high frequency height-oscillations of the shown area. The size of section **a** is $\approx$ 50 µm × 100 µm and was measured with a 150× objective whereas the sizes of **b** and **c** are $\approx$100 µm × 250 µm which were measured with a 50× objective in confocal mode.

Additionally, the influence of the polarization was investigated. Hereby, three different types of laser light polarization were chosen: circular polarized, parallel (oscillation of the electric field along the main writing direction) and perpendicular polarization, respectively. To determine the surface roughness, the obtained image from the optical profilometer was cropped to avoid the influence of missing points. Single sections are depicted in Fig. 4 and the full profilometer images in Supplementary Fig. 2, showing the influence of the polarization at the etched surface of the structured glass.

**Fig. 5:**
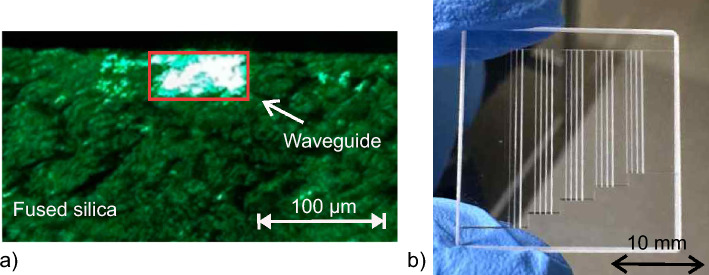
**a** Cross sectional view of the end facet of a 100 µm × 50 µm waveguide with a length of 25.4 mm. The incoupling wavelength was 520 nm. **b** Laser processed sample with different channel lengths for attenuation measurements before etching.

The side wall roughness corresponds similarly to the slicing distance as the roughness on the bottom relies on the hatching distance. The roughness increases with the distance between each structured layer which, however, reduces the production time. After minimizing the surface roughness of SLE written channels, we investigated if waveguiding is possible within the fabricated structures. Therefore, the waveguides were imaged with the setup depicted in Fig. 2. An exemplary cross section image of a 100 µm x 50 µm waveguide with a length of 25.4 mm is shown in Fig. 5a.

The length of the waveguide in this experiment is currently limited by the dimensions of the fused silica substrate. The incoupling and subsequent attenuation measurements were performed with the setup depicted in Fig. 2. The attenuation measurement of the SLE-processed waveguides are not trivial because standard techniques such as the cut-back method are not applicable due to the fused silica substrate. To overcome this issue, we designed a sample with different waveguide lengths and widths on one substrate as shown in Fig. 5b. Additionally, a foldline was added during the SLE process enabling access to the end facets of the waveguides. Thus, the section containing the waveguides can easily be separated from the remaining substrate after the etching. Subsequently, the waveguides were using the setup shown in Fig. 2. Hereby, attenuation values of 0.5 dB/cm, 0.1 dB/cm, 0.34 dB/cm and 0.54 dB/cm with the corresponding waveguide widths of 50 µm × 50 µm, 100 µm × 100 µm, 150 µm × 150 µm and 200 µm × 200 µm, respectively, were obtained. The obvious variations are mainly due to the end facet preparation. At the point where channels are filled with core material, both channel ends are open. This leads to imperfect and different end facet shapes for the manufactured waveguides resulting in varying coupling losses and scattering into the substrate.

**Fig. 6:**
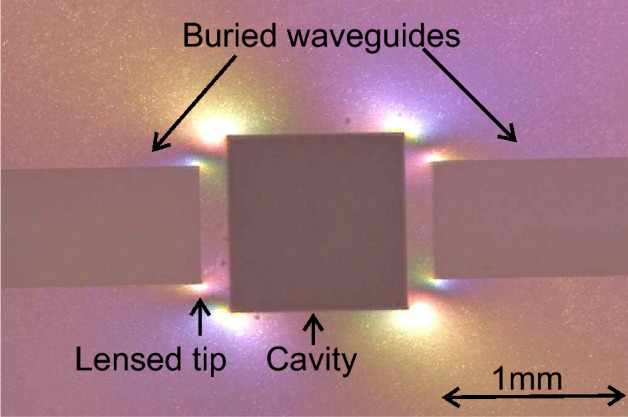
Top view of a structure containing two buried lensed waveguide channels with a cavity in between. The image was taken after the writing process and before the etching using a digital microscope containing polarizer and analyzer orientated perpendicularly to each other. The curvature of the lens is perpendicular to the imaging direction and is not visible in this top-view.

The effect of the polarization is not limited to the surface roughness. This parameter has a significant influence to the internal tension of the structured area which might lead to crack formation. The structure shown in Fig. 6 consists of two waveguiding elements with an elliptical lens at the end which are completely buried within the fused silica. Hereby, the lens is focusing an incoming white light beam along the whole width of the cavity which contains the transparent sample material. The opposite waveguide collects the transmitted light and propagates it to an attached spectrometer. This setup enables a heating of the investigated material with a Peltier element on the bottom side of the glass sheet and an enclosure, by placing an additional glass slide on top to reduce the contact with the ambient air. To enable the etching, the waveguides were angled at $72^{\circ }$ on the opposite side which allows incoupling of the laser light via total internal reflection and connects the buried structure with the surface.

**Fig. 7:**
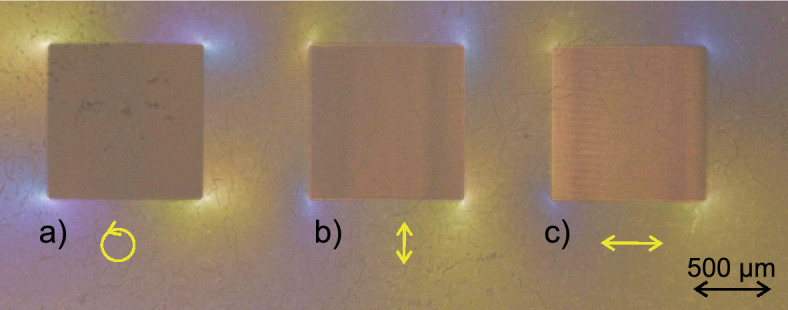
Top view of three squared structures with a size of 1 mm x 1 mm each, obtained by using a **a** circular polarized, **b** linear polarized (in x-direction, parallel to the writing direction) and **c** linear polarized light beam, (in y-direction, perpendicular to the writing direction), respectively. The yellow arrows visualizing the polarization direction.

The squared structure in the middle represents a cavity which can be filled with sample material for transmission measurements. The gap between the waveguides and the cavity is 250 µm. The imaging was done with a digital optical microscope (Keyence, VHX-7000, Germany) containing a polarizer and analyzer in nearly perpendicular orientation. The image shows the tension between the cavity and the waveguides due to tension induced birefringence. The structure which is depicted in Fig. 6 was fabricated using a circular polarized laser beam. Further structures which were written with linear polarization in x or y direction, respectively, resulted in cracks leading to a fracture of the whole structure. An additional investigation of the induced tension regarding the different polarizations is depicted in Fig. 7. The shown modified areas were structured without subsequent etching step to visualize the applied stress depending on the chosen polarization of the writing laser. The other fabrication parameters were similar, i.e. writing speed $v=15\,$mm/s, $P_{avg}=250$ mW and hatching and slicing were set to 1 µm, respectively.

The comparison of the three different polarization modes in Fig. 7 shows that the circular polarized laser beam induces only tension on the edge area whereas the linear polarizations also induce tension on the bottom area, depicted by the higher optical transmission through the squares allthough the integrated polarizer and analyzer are in perpendicular orientation. The structure in Fig. 7b was obtained with a polarization parallel to the writing direction and c) to the polarization perpendicular to the writing direction.

### Microstructuring

**Fig. 8:**
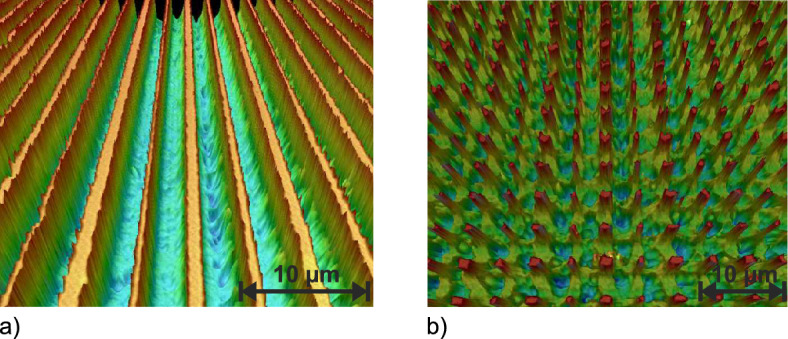
3D-false colour images of the gratings fabricated with the SLE process. The maximum height difference of **a** the line grating is 1.6 µm whereas **b** the 2D-grating shows a height difference of 3.6 µm.

To realize microstructures with SLE, the same process steps as described above are employed. Only the hatching parameter was increased to avoid an overlapping of neighbouring structured lines while minimizing the distance between them. This enables the fabrication of structures with a feature size of less than 1 µm. If this process is repeated along the second axis, single pillars of the same size can be created. The fabricated structures are depicted in Fig. 8.

The smallest feature width of the structures shown in Fig. 8 is $\approx$ 600 nm with an aspect ratio of $\approx$ 2.7. The achieved period of the line grating is $\Lambda =5.1\,$µm ± 0.3 µm. The average feature width is $w_{line}=1.3\,$µm ± 0.3  µm. The corresponding values for the 2D-grating of Fig. 8b are $\Lambda _X=5.1\,$µm ± 0.5 µm with an average $w_X= 1.56\,$µm ± 0.5 µm along the x-axis and $\Lambda _Y=5.1\,$µm ± 0.2 µm with an average $w_Y=1.3$ µm ± 0.2 µm along the y-axis, respectively. The fabricated grating structures were also investigated optically using a HeNe-laser ($\lambda =632.8\,$nm, Melles Geriot, IDEX Health & Science, Carlsbad, Canada). The evaluation of the measured line grating lead to a diffraction angle of $7.2^{\circ }$ of the first diffraction order which corresponds to a grating period of 4.9 µm. The measured values for the 2D-grating are $\Lambda _X=5,2$ µm and $\Lambda _Y=5.34$ µm, respectively. More detailed information regarding characterization of the microstructures are presented in the supplementary material (Supplementary Fig. 3–5).

### Further results towards photonic components created by SLE

**Fig. 9:**
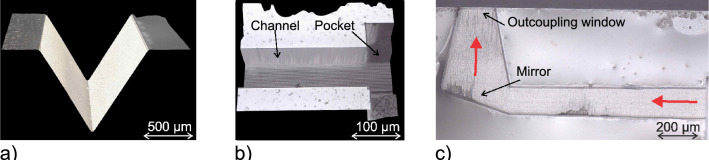
Microscopic images of different structures fabricated using an SLE process. The shown structures can be used for different tasks on a photonic ship. The V-groove in **a** can be designed in a way to place a fiber exactly in front of a subsequent waveguide. The structure shown in **b** enables the placement of a laser diode in front of a waveguide while **c** is a cross sectional view of a buried waveguide with an attached vertical outcoupling element enabled by total internal reflection.

Due to the flexibility of the described SLE process various optical structures were realized. As shown in Fig. 6, it is possible to fabricate buried waveguides without contact to the environment and with an integrated lens at the tip. Additional cavities for spectroscopic analysis can be added as well as microfluidic channels. Supporting structures enabling coupling from a fiber to a waveguide (i.e. printed by two-photon polymerization) or pocket structures with attached waveguide channels for placing micro-filters were produced as well, as shown in Fig. 9. To achieve a low surface roughness for light guiding structures and enabling an exact positioning towards further optical elements, highly accurate stages are required. The system used in this work provides an accuracy of $\approx 300\,nm$ in x-,y- and z-direction (ANT130XY and ANT130LZS, Aerotech Limited, Hampshire, UK).

The combination between a highly accurate process with a large travel range (160 mm on each axis) enables versatile structures to be realized in optical manufacturing.

## Conclusion

Compared to previous work in this field, we focused on the fabrication and characterization of optical structures. Hereby, the focal area and the influence of multiple process parameters were investigated in detail to minimize the surface roughness of the structured area to reduce scattering losses as demanded by applications in integrated optics. The achieved surface roughness of $\approx$ 30 nm enables the fabrication of integrated optical elements. In the next step, more complex and smaller embedded waveguides will be combined to realize integrated optical networks together with micro-fluidic channels. For more complex integrated structures, the used polarization needs to be taken into account due to the possibility that the substrate might be destroyed. The analysis of the interaction of the laser beam with the surface at different z-positions enables the fabrication of even smaller features in the future. Thus, SLE proves a promising technology to realize integrated optics and sensor array by combining additive and subtractive manufacturing technologies. This enables a new field of opto-fluidic sensors by combining customized micro-optics and micro-fluidics within a glass slide.

## Supplementary Information

Supplementary Information.

## Data Availability

All data generated or analyzed during this study are available upon reasonable request from the corresponding author, who is also available to answer questions.
