# Supplementary material for: Surface structuring of glass with submicrometer features using selective laser etching
Source: Sci Rep. 2025 Nov 10;15:39356. doi: 10.1038/s41598-025-27241-0 (PMC12603113; doi:10.1038/s41598-025-27241-0)
Supplement: Supplementary file 1 — Supplementary Information. [file 41598_2025_27241_MOESM1_ESM.pdf]

Supplementary Material:

Surface structuring of glass with submicrometer features using selective laser etching

A. Günther, X. Wang, W. Kowalsky and B. Roth

1. Roughness measurement on manufactured structures

The following images represent the measurement data obtained from the roughness measurements for different polarization states of the writing laser. Data points shown as “0” are invalid values, where the etching did not take place sufficiently, as shown in Fig. 2. The average laser power was increased from 250 mW to 350 mW in 25 mW steps with a repetition rate of the laser at 700 kHz. The hatching distance (distance between each line in the xy-layer) was increased in steps of 0.2 µm, starting from 0.2 µm till 1.0 µm and the slicing distance (distance between each layer along the z-axis) was increased from 1.0 µm to 5.0 µm in steps of 1.0 µm, respectively.

Circular polarization:

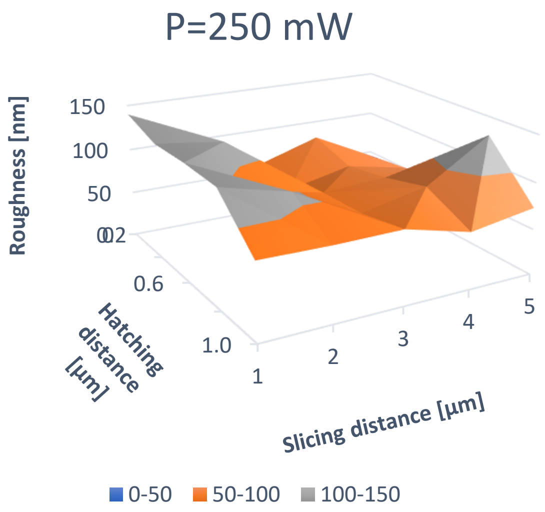

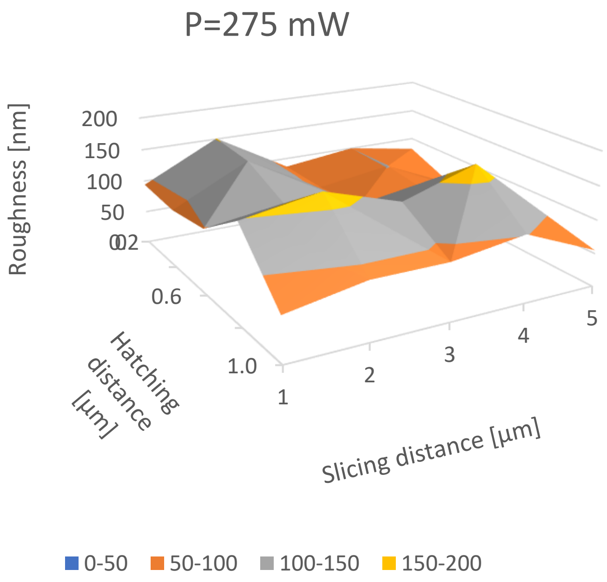

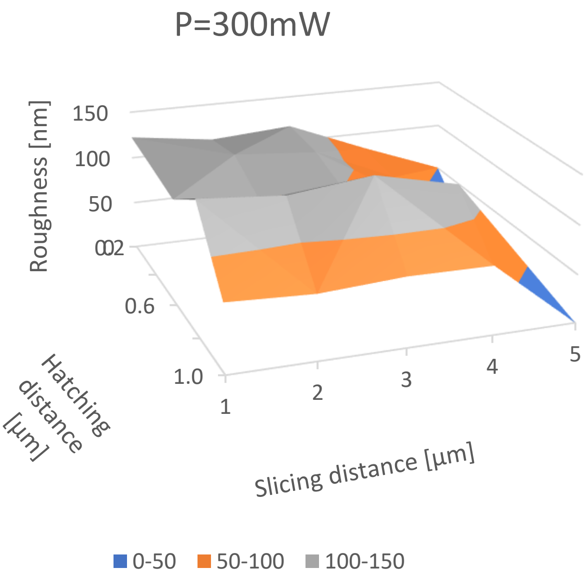

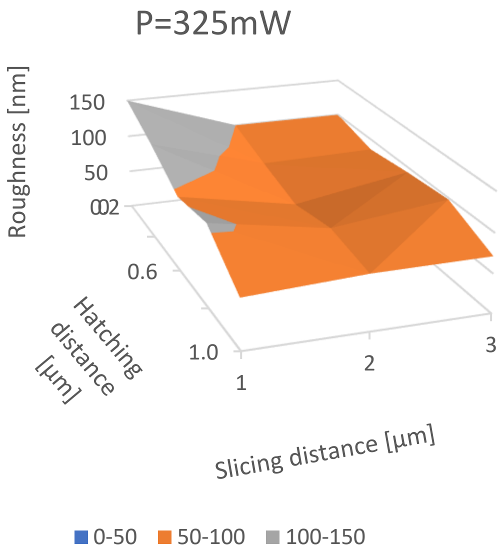

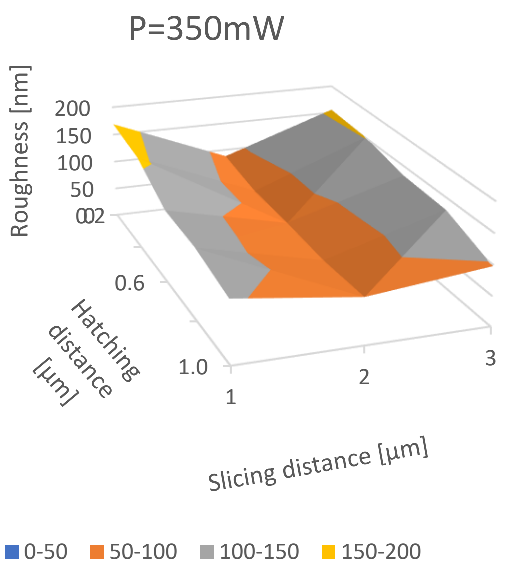

Perpendicular polarization:

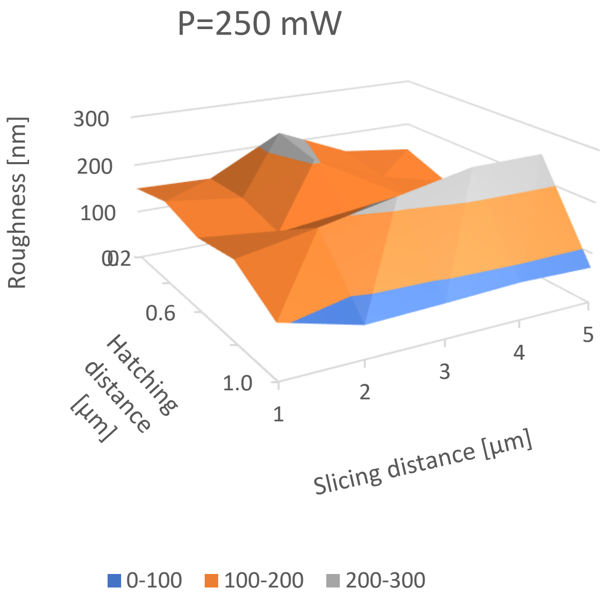

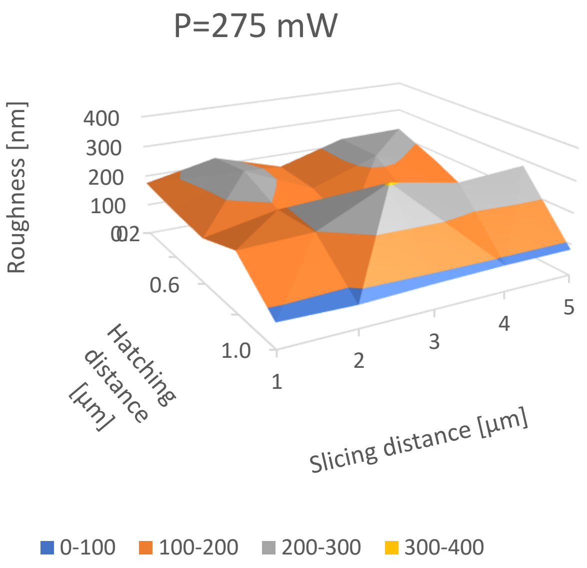

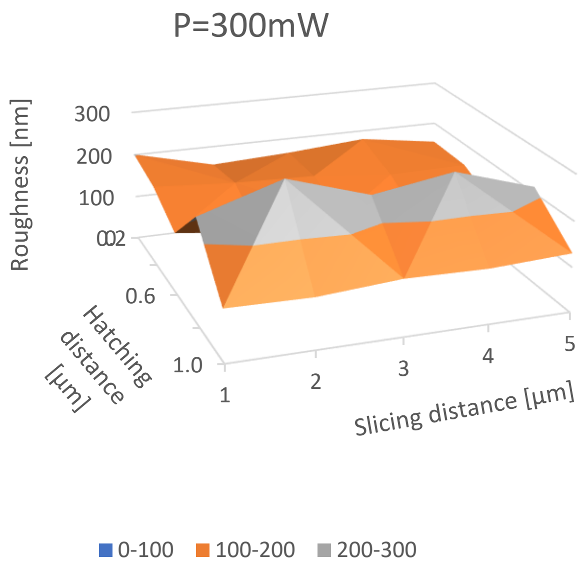

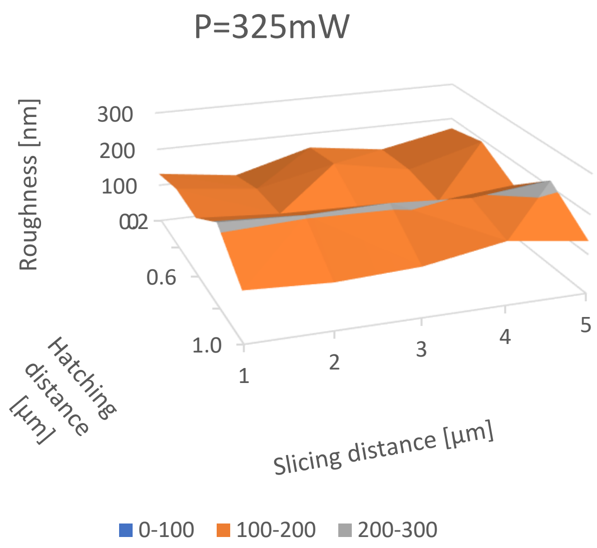

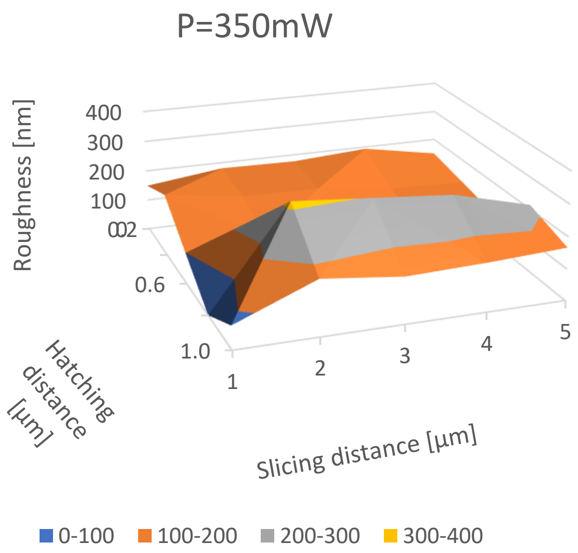

Parallel polarization:

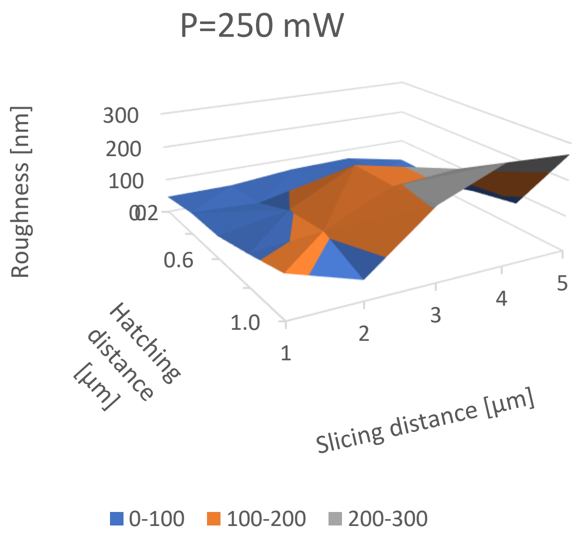

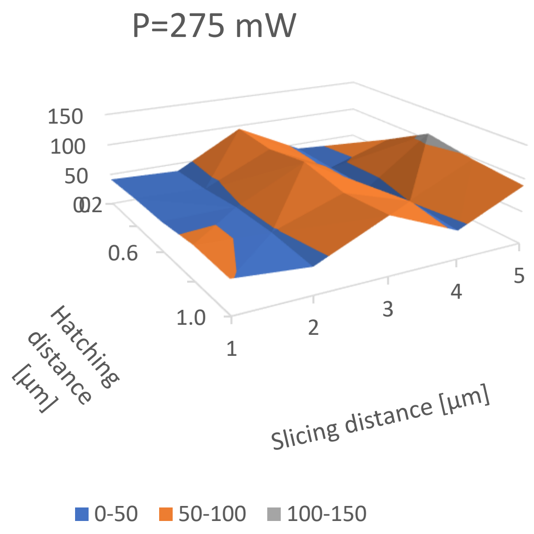

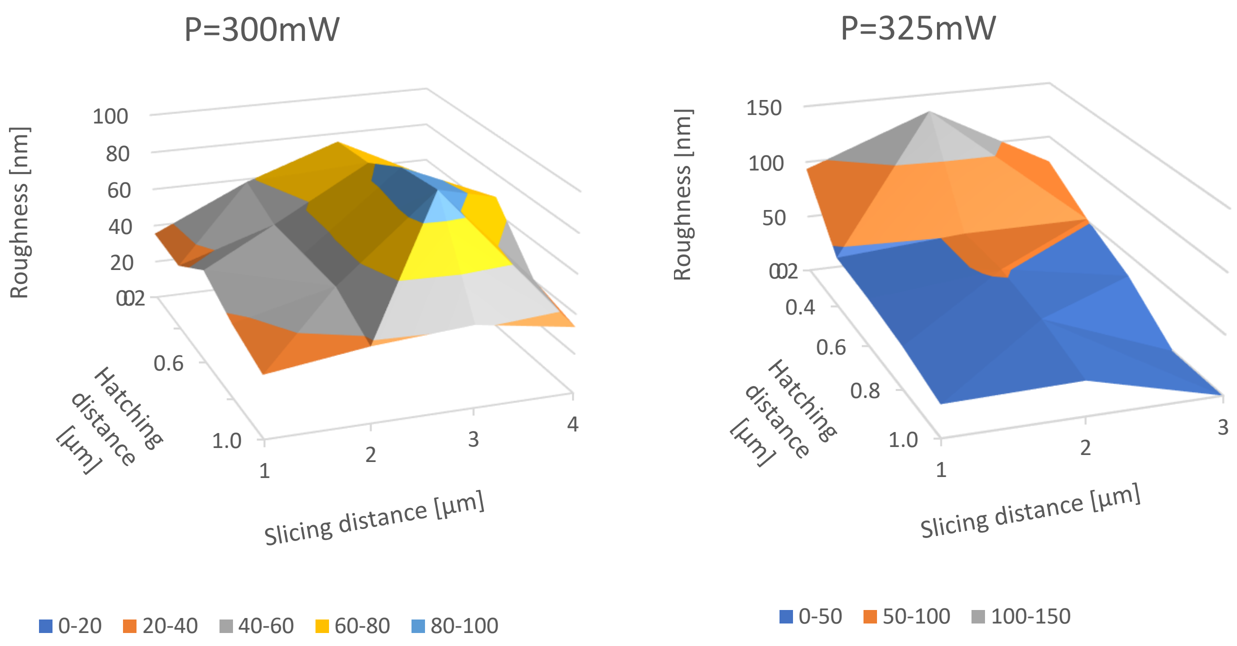

Supplemental Figure 1: Roughness measurement performed with an optical profilometer (Sensofar S neox) as function of different hatching and slicing distances for multiple average laser power polarization settings of the writing laser.

The optimal results for the the surface roughness measurements and the process utilized currently were obtained for parallel polarization and a laser power of 250 mW. In previous experiments, lower values for the slicing were used too. The bottom layer is only affected by the first few layers of the lasing process and the results lead got better if the slicing distance was set > 1µm. The obtained roughness values correspond to values for the bottom surface of the structure. Single measurements were performed to obtain the side wall roughness because it is more difficult to perform measurements there. The obtained results fit well with the roughness data for the bottom layer if the slicing parameter is exchanged with the hatching which means that the slicing distance is the critical parameter for the side wall roughness.

All these measurements were performed at a repetition rate of 500 kHz – 1MHz with 100 kHz steps. The best results were obtained at 700 kHz.

The manuscript contains cropped images of some structure showing the best results for the surface roughness measurement for different polarization settings. The full images are shown in Supplementary Fig. 2.

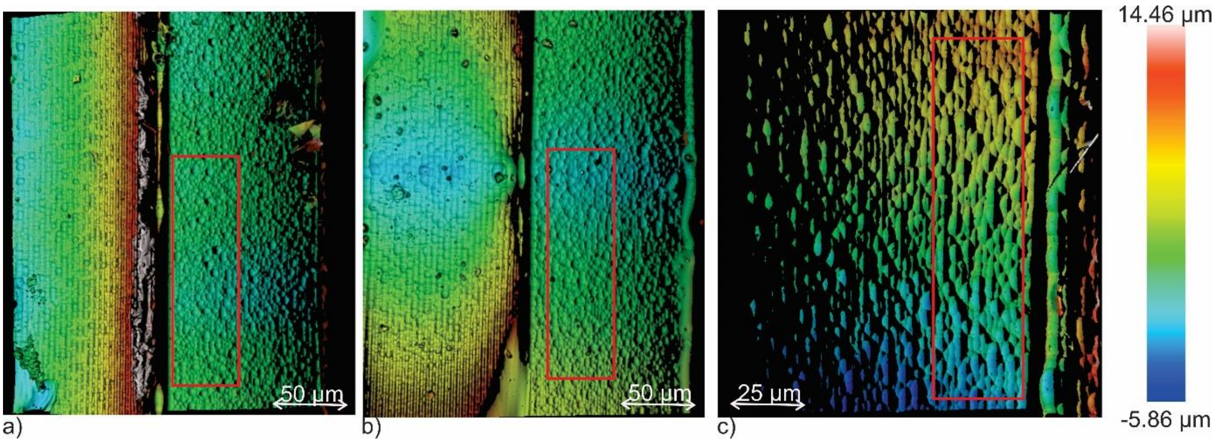

Supplementary Figure 2: Full profilometer images corresponding to the results shown in the manuscript with a) circular, b) perpendicular und c) parallel polarization of the writing laser.

The measurements for perpendicular and circular polarizations were done using a 50x objective. Due to a lack of measurements points based as consequence of low light scattering on the surface, the measurement for the fabricated structure with parallel polarization was done using a 150x objective. The images with the lower magnification also show the neighboring structure written with a different hatching distance.

2. Characterization of manufactured grating structures

The manuscript contains cropped 3D false colour images of the processed grating structures. The full images including a profile line are shown in Supplementary Fig. 3.

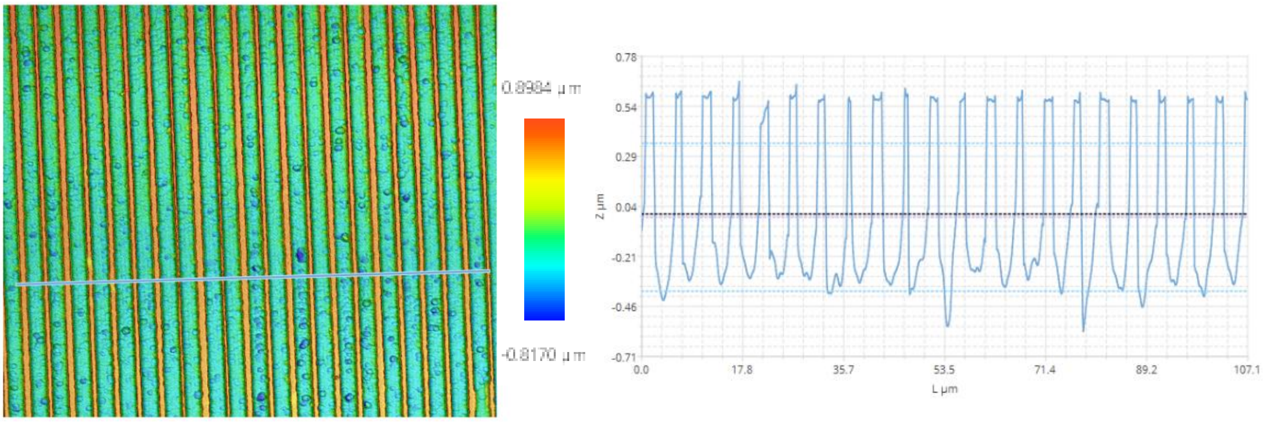

Supplementary Figure 3: Image of the SLE processed grating structure with the corresponding profile line.

The corresponding full image of the 2D grating is shown in Supplementary Fig. 4.

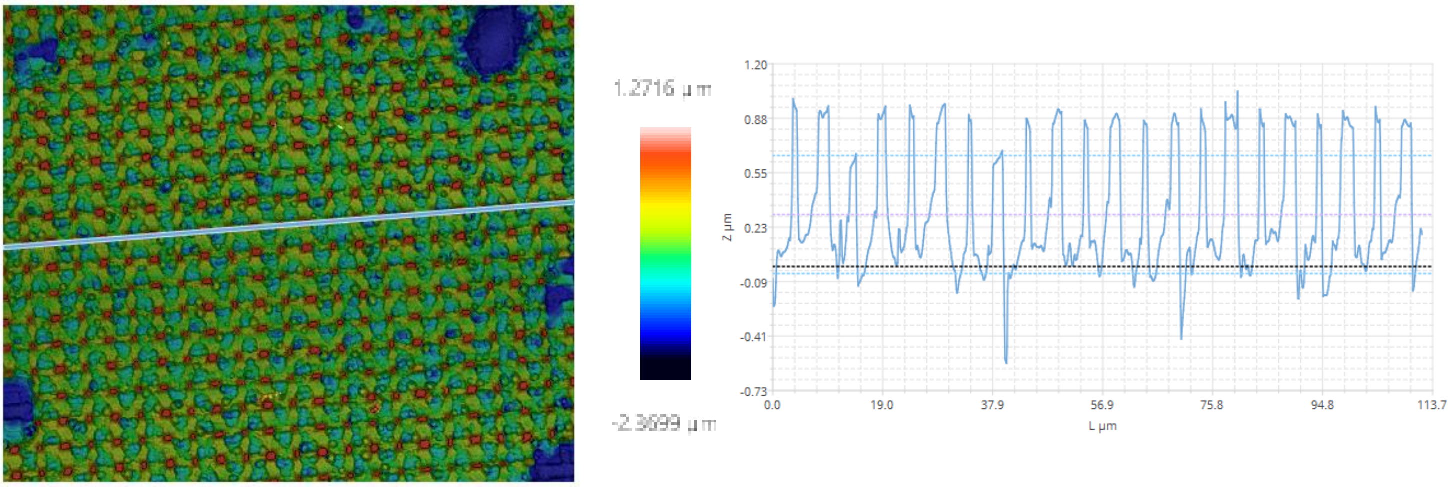

Supplementary Figure 4: Image of the 2D grating with corresponding profile line.

For the optical characterization of the grating structure a HeNe laser was used. The resulting diffraction patterns are depicted in Supplementary Fig. 5.

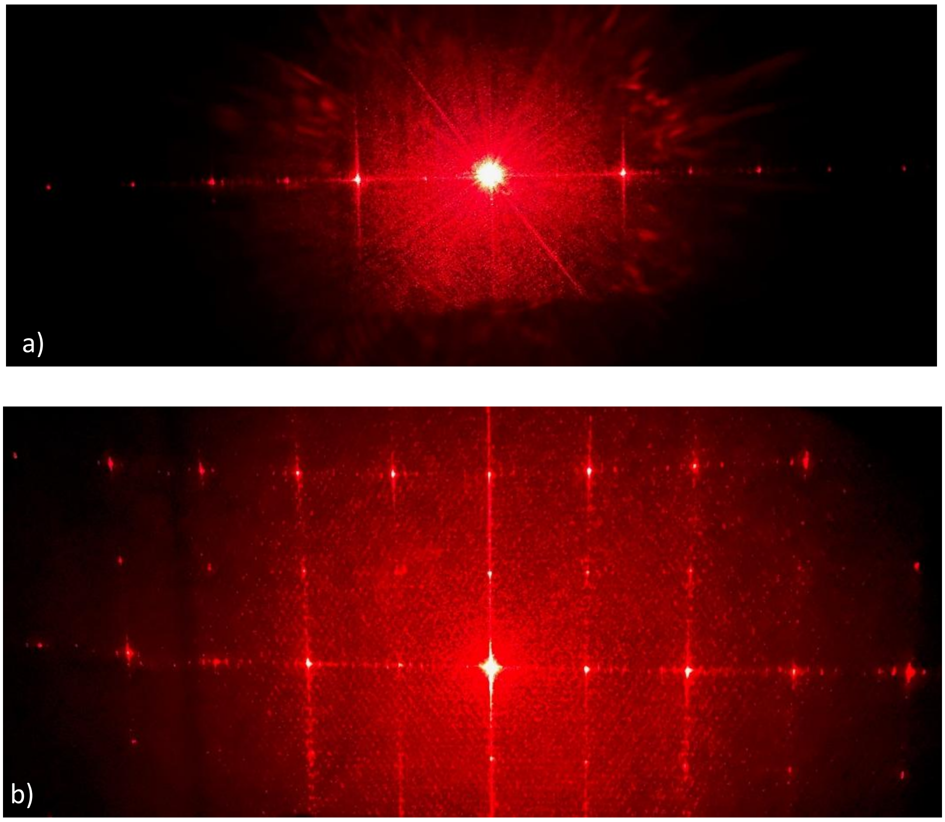

Supplementary Figure 5: Obtained diffraction patterns by illuminating the a) line grating and b) 2D grating with a HeNe-laser.
